# Plastic Dentistry

**Published:** 1877-07

**Authors:** H. S. Chase


### PLASTIC DENTISTRY.

BY H. S. CHASE. D. D. L.

*Read before the Indiana State Dental Association.*

I believe the coming dentist will be one who will use plastic materials almost exclusively for filling teeth. The tendency of thought is in that direction to-day. In a few years you shall look back upon the operations of to-day as clumsy and unscientific. The need of to-day is a white plastic material which will be more in harmony with tooth substance than any thing that we have at present. In color, gold is not in harmony with dentos, but it is still more unharmonious in its chemical and galvanic relations. A tooth containing a gold plug is attacked, so to speak, more savagely by a weak acid than a similar tooth in which there is no plug at all.

The presence of the gold invites the attack.

Experiments prove this and confirms a well known law of galvanic action. The same may be said of all our alloys and amalgams, and of tin also. The latter excites chemical action between a weak acid and dentos in a less degree than either gold or our many alloys and amalgams. However between tin and amalgams containing much tin, there is little or no difference. The quicksilver used in making amalgams containing gold, silver and tin reduces their conductivity and also makes them less electro-negative.

These are two important points in a filling material. It must be remembered that dentos is very electro-positive, as is proved by its easy disintegration in a weak acid. It is necessary that we bring our filling material as near dentos on the electro-positive scale as safety will allow. Ox. chlo. zinc is very near—in fact too near.

The positive of gold as an electro-negative makes it very dangerous in the presence of a weak acid. This is one of the reasons why so many thousands of gold fillings fail in a few years. Another reason is that so many gold fillings do not form a water-tight plug. When the latter is the case the electro-negative character of gold causes a tenfold acceleration of chemical or galvanic disintegration. Whether this action is a true electrolysis or not, the baneful result is the same, even if it is entirely chemical. It is certainly very painful to an honest dentist to find out suddenly that gold is not the best material with which to plug decayed cavities. When the operator becomes convinced of this his next enquiry will be for something better. When this want is felt, thought will be stimulated to produce better results. We have not advanced very far toward the goal yet. Tin is sufficiently preservative, but its color is not desirable, and it is too soft for masticating surfaces. Amalgams cannot boast of color over tin, but they can on account of hardness. The greater number of alloys for amalgams in the market have a positive defect in contraction after they are in the cavity, thus causing leakage.

Although a leaky amalgam plug is not as pernicious as a leaky gold one, yet it should and may be entirely avoided.

Amalgams can be used which do not contract in hardening. Such amalgams are easily manipulated into a water-tight plug.

Some amalgams discolor too easily in the mouth for a pleasant appearance. Those which contain the most tin and gold, and the least silver keep a good color far better than those with larger proportion of silver. The difference is indeed very marked.

If you ask me this question, what do you fill cavities in molar and bicuspids with? My answer is this. With an amalgamated alloy containing about 25 parts gold, 39 parts tin, and 36 parts silver, requiring from 30 to 50 parts of mercury.

In some proximal fillings I use 50 parts tin. The more tin the whiter the plug, but of less strength. No alloy should be used as a plug until that particular sample has been subjected by the operator to the tube test for leakage.

A leaky plug is an abomination. How many operators have tried their gold plugs by the tube test?

Let dentists measure themselves in this respect. For the present I know of no full substitute for gold in the front teeth. Sometimes the gold and tin alloy may be used to good advantage.

Where plugs show I still use gold. If gutta percha stopping did not contract it would do very well until something better is discovered. But some gutta percha material loses too much by chemical disintegration in some mouths. At present I know of no gutta percha stopping that will stand the tube test.

However a leaky gutta percha plug will not cause dentos to decay as gold does, owing to its low conductivity. Gold is very negative and very conductive ; these two qualities make it worse than gutta percha. In a scale of 100: Silver is 100; Gold is 60; Tin is 30: Amalgam is 10 to 20; Gutta Percha 13. As to negativity: Gold is 100; Amalgam 65; Tin 60; Gutta Percha filling 50; Ox chlo. zinc, 45. I wish no one to adopt my views unless they appear reasonable to him. I wish none to condemn them until after a large amount of experimental work and study.

And to induce the latter is the object of this paper.

HENRY S. CHASE, M. D.

